# HUMESS: integrating quantitative transcriptomic analysis and metabolic modeling to unveil condition-specific gene signatures

**DOI:** 10.1093/bioinformatics/btaf448

**Published:** 2025-08-12

**Authors:** Louis Paré, Philippe Bordron, Laurent David, Maxime Mahé, Audrey Bihouée, Damien Eveillard

**Affiliations:** Nantes Université, Centrale Nantes, CNRS, LS2N, Nantes 44322, France; Université de Toulouse, INRAE, BioinfOmics, GenoToul Bioinformatics Facility, Castanet-Tolosan 31320, France; Université de Toulouse, INRAE, UR 875 MIAT, Castanet-Tolosan 31320, France; Nantes Université, Inserm, CR2TI, Nantes F-44000, France; Nantes Université, CHU Nantes, CNRS, Inserm, BioCore, Nantes F-44000, France; Nantes Université, Inserm, TENS, The Enteric Nervous System in Gut and Brain Diseases, IMAD, Nantes 44000, France; Center for Stem Cell and Organoid Medicine (CuSTOM), Cincinnati Children’s Hospital Medical Center, Cincinnati, OH 45229,United States; Department of Pediatrics, University of Cincinnati, Cincinnati, OH 45267-0515, United States; Nantes Université, CNRS, INSERM, l’institut du thorax, Nantes F-44000, France; Nantes Université, Centrale Nantes, CNRS, LS2N, Nantes 44322, France

## Abstract

**Summary:**

Transcriptomic analysis is a key tool for exploring gene expression, but the complexity of biological systems often limits its insights. In particular, the lack of intermodal or multi-layered analysis hinders the ability to fully capture key cellular functions such as metabolism from transcriptomic data alone. Here, we introduce a novel approach that informs transcriptomic data analysis with metabolic network modeling to address this. Unlike traditional methods, HUman MEtabolism Specific Signature (HUMESS) uses genome-scale metabolic modeling and flux analysis to highlight reactions and involved genes based on their metabolic significance, offering a deeper understanding of transcriptomic data. Our computational pipeline, supported by a user-friendly Rshiny application, enhances gene expression analysis by uncovering metabolic phenotypic signatures.

**Availability and implementation:**

HUMESS is open source and available under GitLab https://gitlab.univ-nantes.fr/bird_pipeline_registry/humess with the complete documentation available at https://gitlab.univ-nantes.fr/bird_pipeline_registry/humess/-/wikis/Home. A zenodo archive is also available at the following DOI: https://doi.org/10.5281/zenodo.15487717. An RShiny application has been developed to facilitate the exploration and analysis of HUMESS’s results. The app is available online at the following address: https://shiny-bird.univ-nantes.fr/app/shinymess but can also be installed locally, available under GitLab https://gitlab.univ-nantes.fr/pare-l/shinymess.

## 1 Introduction

Transcriptomic analysis is a powerful tool for elucidating gene expression patterns associated with specific biological conditions, offering invaluable insights into cellular responses and regulatory mechanisms (Melé *et al.* 2015). However, despite its utility, many transcriptomic studies fail to provide comprehensive insights due to the inherent complexity (i.e. multi-layered omics intricacy) of biological systems and the limitations of purely expression-based approaches. One major challenge in transcriptomic analysis is the need for external knowledge to interpret gene expression changes in a meaningful biological context, which is labor-intensive and prone to biases (Khatri *et al.* 2012). Consequently, many gene expression signatures derived from transcriptomic data remain superficial, and lack the depth necessary for proper mechanistic understanding.

Whereas conventional methodologies perceive alterations in gene expression in isolation from one another, contemporary approaches endeavor to consolidate genes into extensive collections for the evaluation of phenotypic characteristics. For example, network analyses advocate for the aggregation of genes predicated on their co-expression (Langfelder and Horvath 2008) or the association of genes based on their mechanistic activation or repression (Karlebach and Shamir 2008). More recently, initiatives have been directed toward quantitative biology to evaluate metabolic networks. These networks exemplify a collection of reactions that are encoded by gene products and are interrelated when the output of one reaction serves as the substrate for another. Recent advancements have facilitated the conversion of these networks into genome-scale metabolic models (i.e. GEMs) to evaluate the fluxes executed by all metabolic reactions (Gu *et al.* 2019). The assessment of such quantitative insights encapsulates metabolic phenotypes and is particularly applicable to human data, as it encompasses the most comprehensive dataset of multi-modal information. Integration of diverse omics data with GEMs continues to pose a significant challenge, as it necessitates the harmonization of information from multiple sources to reflect the intricacies of human metabolism accurately. The integration of omics data with GEMs has been achieved through several methods [see (Moškon and Režen 2023) for review]. However, this integration will most often necessitate the collection of additional tedious information (i.e. in-house objective function) or rely on computational solutions that are not open-source. Furthermore, the resulting GEMs often pose a challenge for users who want to make the most of their transcriptional data and are not necessarily familiar with genome-scale metabolic model analysis.

Here, we introduce a novel approach termed HUman MEtabolism Specific Signature (HUMESS), which seeks to bridge this gap by leveraging recent advances in quantitative transcriptomic analysis and metabolic modeling. The uniqueness of this research is founded upon the application of methodologies originally devised for the modeling of bacterial GEMs (Machado *et al.* 2018). Rather than analyzing gene expression individually or via correlative approaches, a systematic analysis of mechanistic dependencies between metabolic reactions is performed to be then propagated on associated genes.

By integrating metabolic modeling results with transcriptomic analysis, HUMESS provides a unique framework for interpreting Human quantitative transcriptomic data without requiring additional knowledge. It highlights genes and pathways that are mechanistically linked and, among them, identifies those essential to support a metabolic phenotype in specific conditions. This integration allows the refinement of gene lists based on their metabolic significance, providing a more nuanced understanding of cellular responses and holistic metabolic implications.

This manuscript describes a computational pipeline that uniquely combine RNA data and metabolic model reconstruction in Python to perform analysis. For a user friendly interface, the pipeline is accompanied by a Rshiny web application (Chang *et al.* 2012) that permits loading pipeline output files for manipulating various graphical representations (i.e. MA and Volcano plots). We demonstrate the utility of HUMESS by improving the analysis of recent case studies in human development (Onfray *et al.* 2024). By elucidating condition-specific gene signatures weighted by their metabolic relevance, we showcase the power of integrating quantitative transcriptomic analysis and metabolic modeling to uncover novel insights into biological systems. Through HUMESS, we aim to provide a user-friendly, valuable tool that augments gene expression data with a metabolic scope, eliminating the need for prior knowledge of metabolic analysis, and ultimately advancing our understanding of complex biological phenomena.

## 2 Combining transcriptomic analysis and human metabolic modelings

**Figure 1.**
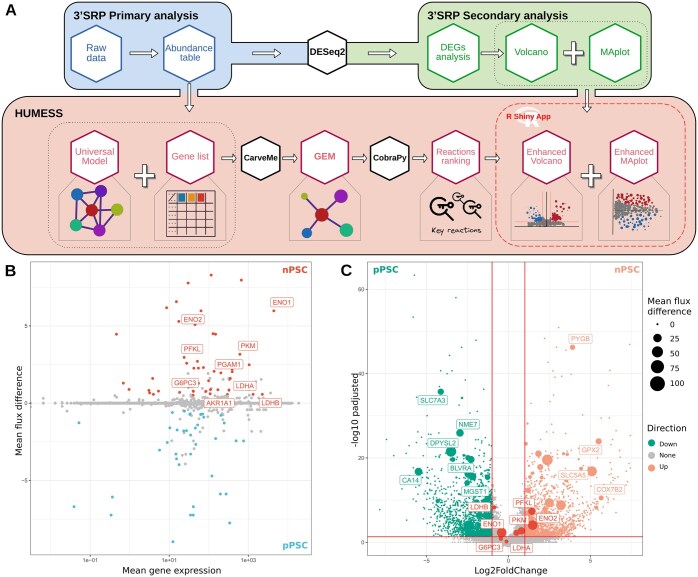
Description of the HUMESS pipeline and associated representations. (A) HUMESS feeds on standard outputs of transcriptomic analysis to build a GEnome-scale Metabolic (GEM) model via a top-down approach. Sampling analyses are then performed to identify the reaction cumulative correlation score (RCC) and mean flux per GEM’s reactions. A modified logo of R is used, from The R Foundation https://creativecommons.org/licenses/by-sa/4.0/. (B) Projection of mean gene expression against the differential of mean flux values of associated metabolic reactions between the nPSC and pPSC conditions. Genes implicated in the glycolysis and glyconeogenesis pathway are labeled. (C) Volcano plot comparing the nPSC and pPSC conditions with the size of nodes proportional to the absolute value of the difference of mean fluxes between conditions. Genes implicated in the glycolysis and glyconeogenesis pathway are highlighted.

HUMESS has been developed using the 3’SRP pipeline for transcriptomic analysis as input (Charpentier *et al.* 2021). In contrast to conventional RNA-seq, 3’SRP analysis has the advantage of multiplexing samples and barcoding transcripts with single molecule identifiers (UMIs), significantly improving the accuracy of mRNA quantification. The HUMESS pipeline embeds gene-expressed data within a metabolic modeling framework for reconstructing a metabolic network of the human systems analyzed. Beyond the sole availability of the context-specific metabolic model, HUMESS will conduct a systematic exploration of reaction fluxes to highlight essential features. This information will then be used to enhance the transcriptomic analysis and aid in identifying phenotypic biomarkers. The pipeline follows the steps below illustrated in Fig. 1A.

### 2.1 Reconstruction of human metabolic models from expressed genes

HUMESS uses UMI count tables to feed an adaptation of a top-down metabolic network reconstruction, called CarveMe (Machado *et al.* 2018). This protocol consists of *carving* the universal metabolic model to fit genes expressed in the given condition while maintaining functionality (i.e. ensuring a maximum amount of flux through the biomass reaction). Initially developed on prokaryotes, we improved CarveMe to (i) use a genome-scale human meta-model, called Recon3D (Brunk *et al.* 2018), obtained from the BIGG (King *et al.* 2016) database, that summarize high-quality, manually curated metabolic constraints associated to Human; and (ii) use a list of expressed genes instead of a genome. Since only the information of which gene is supposedly present is used, a particular attention must be given to the gene expression thresholding. HUMESS implement several filters, as described in the documentation. By default, we recommend the use of filterByExpr from the edgeR R package (Robinson *et al.* 2010).

### 2.2 Exploration of the metabolic model solution space

The resulting context-specific metabolic models incorporate all stoichiometric and thermodynamic constraints that one aims to solve under mass action law and quasi-steady state conditions. This leads to a solution space for each context-specific model that we explore through the COBRApy library (Ebrahim *et al.* 2013), particularly via randomized flux sampling analysis. For this purpose, HUMESS embeds two complementary techniques: a global solution space sampling via OptGPSampler (Megchelenbrink *et al.* 2014) and the corner-based sampling (Galuzzi *et al.* 2024). The first technique emphasizes the phenotype in exploring the whole solution space. This is computationally challenging, especially for large numbers of samples and a high thinning rate (i.e. reducing redundant data points). The second approach focuses on exploring the boundaries (corners) of the solution space. Therefore, this exploration weighs more quickly on extreme behaviors of the metabolic phenotype. The limits of sampling the solution space are undersampling (i.e. the lack of convergence) or oversampling (i.e. magnified autocorrelation). HUMESS avoids these limitations by using Raftery & Lewis and Geweke’s convergence tests (Fallahi *et al.* 2020) to find the optimal number of samples for exploring solutions.

### 2.3 Identification of essential reactions and associated genes

Using the sampling results, pairwise correlation is computed for each metabolic reaction against every other reactions, resulting in a symmetric correlation matrix. We define each reaction’s Reaction Cumulative Correlation score (RCC) as the sum of all pairwise correlations, in absolute value, associated with the given reaction. This results in a value ranging from 0 to the number of total reactions. Thus, a reaction associated with a high value of RCC, which is highly correlated with all given GEM reactions, is considered essential to sustain metabolic phenotypes, as any flux alterations on this particular reaction will impact all others. A distribution of RCC (i.e. histogram) or a list of all reactions ranked based on RCC assesses a comparison between different metabolic models. Furthermore, the mean value of all fluxes ($mol.kgDW^{-1}.h^{-1}$) sampled per reaction is calculated. Worth noting, mean value of all metabolic fluxes reflect the reaction usage (i.e. amount of metabolic material passing through), whereas RCC reflect the importance of the reaction in maintaining the metabolic phenotype (i.e. weighted centrality).

Using the Gene–Protein–Reaction rules obtained from the BIGG database, RCC and usage of reactions is mapped to the genes involved in those reactions. HUMESS incorporates these scores in standard transcriptomic visualizations to help highlight essential genes in the metabolic network. An MA-plot-like visualization (see Fig. 1B) describes each GEM’s gene based on its expression compared to its associated reaction importance score. Similarly, conditions are compared using Volcano plots, incorporating the differential reaction importance score between the two given GEMs using the node size (see Fig. 1C). These representations are the first step in incorporating results from metabolic modeling and help highlight genes with strong importance in the metabolic phenotype via an illustration that is standard in transcriptomic analysis.

The results from the metabolic modeling can be further analyzed by selecting reactions and associated genes that are most different in metabolic score between two conditions. Similarly to what can be achieved through differential expression analysis in transcriptomic, enrichment analyses can be applied, such as Over-Representation Analysis (ORA) or Gene Set Enrichment Analysis (GSEA) (Subramanian *et al.* 2005) based on different databases such as Gene Ontology (GO) terms or KEGG pathways solely on the difference in reaction importance as a discriminatory score.

## 3 Magnifying the whole stem cell transcriptomic analysis

To validate our approach, we applied HUMESS to existing transcriptomic data from various stages of human embryonic stem cell development. Specifically, Onfray and colleagues (Onfray *et al.* 2024) examined, using a combination of transcriptomic, proteomic, epigenetic as well as metabolic approaches, several states of pluripotency: (i) naive (nPSC, modeling human epiblast between six to nine days after fertilization), (ii) primed pluripotent stem cells (pPSC, modeling human epiblast between ten to fourteen days after fertilization), (iii) extended PSCs (ePSC, pluripotent stem cells with extended potential) and (iv) trophoblast stem cells (TSC, derived from human blastocyst but also from first trimester placenta). Through their sole transcriptomic analysis, Onfray and colleagues discovered thousand of genes showing differential expression between all these states.

Building on these findings, we developed four types of GEMs for stem cells: the nPSC-like GEM (2087 metabolites, 2933 reactions and 509 genes), the pPSC-like GEM (1961 metabolites, 2692 reactions and 508 genes), the ePSC-like GEM (1900 metabolites, 2583 reactions and 484 genes), and the TSC-like GEM (2495 metabolites, 3773 reactions and 618 genes). Flux sampling was carried out with a thinning value of 10 000 for each of the four GEMs, resulting in sample sizes of 100 154 for nPSC, 104 670 for pPSC, 150 000 for ePSC, and 105 517 for TSC.

We used HUMESS to improve the interpretation of two comparisons based on the transcriptomic data. Firstly, we focused on the nPSCs compared to the pPSCs conditions, in which the metabolic differences have been widely studied (Gu *et al.* 2016). Specifically, we focused on genes with significant differential mean values of fluxes (i.e. *P*-value < .05) exhibiting modifications in associated reaction flux (i.e. above 0.5 for nPSC versus pPSC and below −0.5 for pPSC versus nPSC). In the nPSC versus pPSC scenario, KEGG pathway enrichment analysis revealed that the most enriched pathway is the glycolysis and glyconeogenesis pathway, with 11 genes implicated (ENO1, ENO2, PFKL, PGAM1, PKM, G6PC3, AKR1A1, LDHA, LDHB). Using transcriptomic data alone, only two of them would have been identified, as they were the only one exhibiting a significant expression fold change (> one absolute Fold Change) (see Fig. 1C). Furthermore, the fourth most enriched pathway is the biosynthesis of amino acids. Together, these findings support the metabolic distinctions pointed out by Gu *et al.* highlighting an increase in glycolysis in the nPSC condition; Indeed, their findings, based on metabolic measurements, also highlighted an increased use of glucose for the biosynthesis of nucleotides and serine. The strength of HUMESS resides in elaborating a specific metabolic network for each condition, which can be used to deepen the analysis of the differences between the two conditions, notably by highlighting exactly which reactions are responsible for the shown difference. Here, we could discriminate the phosphoglucomutase reaction, responsible for converting glucose-1-phosphate to glucose-6-phosphate at the very start of the glycolysis process, with a higher metabolic flux in nPSC against pPSC. This reaction is linked to the PGM2 gene, preferentially expressed in nPSC. Two other reactions were also found with higher flux in nPSC toward the end of the glycolysis pathway, the enolase and the pyruvate kinase reaction, which are responsible for the generation of pyruvate.

The ePSC versus pPSC comparison was also studied using HUMESS; the transcriptomic analysis by Onfray *et al.* showed only 180 differentially expressed genes. Using HUMESS, we could discriminate genes that exhibit higher reaction flux in the ePSC conditions and search for enriched pathways. The third most enriched pathway is oxidative phosphorylation, while glycolysis/gluconeogenesis is among the enriched pathways. Both of those findings aligns with metabolic distinctions pointed out by Onfray and colleagues through specific experimental metabolic measurements, the extracellular acidifiation rate that is directly associated with glycolysis, and the oxygen consumption rate, linked to oxidative phosphorylation.

## 4 Conclusion

HUMESS enhances transcriptomic analysis as an independent tool by pinpointing genes particularly involved in the metabolism of a given condition, that expression data alone would difficultly detect. Beyond ranking the gene set, we propose that HUMESS could also inform metabolomics studies by identifying a collection of metabolites anticipated to be produced in excess within particular GEMs. Viewed from a wider lens, the primary aim of HUMESS is to recommend specific GEMs suited for future mechanistic research. We anticipate that this will aid in uncovering genes that maximize specific metabolites and allow for more thorough investigations into the transitions among various cell states, including those related to cell differentiation, as well as the exploration of the entropy associated with biological systems.

## Data Availability

No new data were generated in support of this research. The software is available through gitlab at the following address: https://gitlab.univ-nantes.fr/bird_pipeline_registry/humess and the shiny interface is available at the following address: https://gitlab.univ-nantes.fr/pare-l/shinymess
